# Raman imaging for the analysis of silicone microplastics and nanoplastics released from a kitchen sealant

**DOI:** 10.3389/fchem.2023.1165523

**Published:** 2023-05-17

**Authors:** Cheng Fang, Yunlong Luo, Ravi Naidu

**Affiliations:** ^1^ Global Centre for Environmental Remediation (GCER), University of Newcastle, Callaghan, NSW, Australia; ^2^ Cooperative Research Centre for Contamination Assessment and Remediation of the Environment (CRC CARE), University of Newcastle, Callaghan, NSW, Australia

**Keywords:** silicone sealant, microplastic, nanoplastic, Raman imaging, Gaussian surface, image reconstruction

## Abstract

Plastic products are used ubiquitously and can potentially release microplastics and nanoplastics into the environment, for example, products such as the silicone sealant used in kitchens. It is important to develop an effective method to monitor these emerging contaminants, as reported herein. By using advanced Raman imaging to characterize microplastics and nanoplastics from hundreds of spectra in a scanning spectrum matrix and not from a single spectrum or peak, the signal-to-noise ratio can be significantly increased, from a statistical point of view. The diffraction of the laser spot usually constrains the imaging resolution (such as at ∼300 nm), which is also pushed to the limit in this report by shrinking the scanning pixel size down to ∼50 nm to capture and image small nanoplastics effectively. To this end, image reconstruction is developed to successfully pick up the meaningful Raman signal and intentionally avoid the noise. The results indicate that the silicone sealant in a kitchen can release a significant amount of microplastics and nanoplastics. Overall, advanced Raman imaging can be employed to characterize the microplastics and even nanoplastics that are smaller than the diffraction limit of the laser via Raman imaging and image reconstruction toward deconvolution.

## 1 Introduction

The amount of plastic produced has been increasing continuously over the last few decades, and this production is anticipated to reach over one billion tons annually by ∼2050 (Geyer, 2020). The growth in the use of plastic is due to its beneficial properties, such as versatility, durability, and low cost. However, as a consequence of improper disposal and mismanagement of plastic products, plastic contamination has become a severe global problem. The accumulation of plastic litter in the environment is a growing menace, threatening wildlife and causing damage to the ecosystem. Plastic waste also poses a risk to human health by contaminating food sources and drinking water supplies (Sharma et al., 2022). Although some earlier research focused largely on the negative impact of plastic chemicals, such as bisphenol-A and phthalates, recent studies have reported new problems related to microplastics (1–5 mm) and nanoplastics (<1 µm) (Hartmann et al., 2019; Ivleva, 2021). These tiny plastic fragments have been detected in human blood (Leslie et al., 2022), stools (Schwabl et al., 2019), lungs (Amato-Lourenço et al., 2021), and placenta (Ragusa et al., 2021). Urgent action is required to better understand their sources, fates, pathways, and toxicities.

Microplastics can result from the mechanical, chemical, or biological degradation of large items in the environment, such as via weathering, solar radiation, and microbial degradation (Corcoran, 2020). What is also becoming increasingly clear is that microplastics can also be generated during day-to-day activities. Some research has been performed to investigate how microplastics are created during the process of lawn mowing (Luo et al., 2021), laundering (Henry et al., 2019), and cooking (Luo et al., 2022a). Compared with microplastics found in the environment, those directly released from daily activities have received far less attention, even though they might be more serious as a result of direct human exposure (Ouyang et al., 2022).

Plastic is made of polymers, and each polymer has unique properties. An elastomer is a polymer featuring a high level of viscoelasticity, which can be used in rubbery materials, for example (Wang et al., 2021). It is well recognized that organic elastomer products, such as tires, are a significant source of microplastics (Halle et al., 2020). Tire wear and tear, as a result of abrasion, has been estimated to contribute ∼28% of microplastic pollution in the sea (Bondelind et al., 2020). On the other hand, far too little attention has been paid to other elastomers, such as silicone or polysiloxane, which can also be categorized as plastics (Hartmann et al., 2019).

Silicone is widely used as sealants, adhesives, lubricants, insulants, and cooking utensils, and is commonly found in the kitchen. Silicone sealants are useful in sealing kitchen benches around the edges, preventing water from entering the space underneath the covering. Although the use of silicone products is generally safe, an earlier investigation has reported the occurrence of silicone microplastics in wastewater treatment plant effluent and aquatic ecosystems (Fan et al., 2022). These initial findings indicate a need to better monitor and source silicone microplastics and potentially more hazardous nanoplastics. Once released in the kitchen, they can lead to direct human exposure via food contamination (during food preparation) or environmental pollution via sink to wastewater treatment plants and eventually pose a risk to aquatic ecosystems (Zhao et al., 2015; Hartmann et al., 2019; Oliveri Conti et al., 2020).

Microplastic and nanoplastic quantification is challenging due to several reasons including the small size of particulate matter, the diversity of the shapes/components, and the complexity of the environmental samples (Ivleva, 2021). The small particulate matter (not ion and not simple molecule) creates significant difficulties in extraction and sample preparation because traditional approaches might not work here, particularly for nanoplastics. Microplastics and nanoplastics come in a variety of sizes and shapes, including fibers, fragments, beads, and other irregular shapes with different components (polymer and additives), which can complicate the detection process (Shruti et al., 2021). Collecting representative samples from the environment can also be difficult due to their low abundance, the heterogeneity of environmental matrices, and the presence of coexistence (e.g., bio and mineral dust) that can interfere with the analysis (Ruggero et al., 2020). The lack of standardized analysis methods makes it difficult to assess the risk of microplastics and nanoplastics (Hermsen et al., 2018). Although research is ongoing, some progress has been achieved in the last few decades.

Raman imaging can be an effective approach to monitor and characterize silicone micro- and nanoplastics, by analyzing the unique spectrum containing characteristic peaks like fingerprints (Österle et al., 2015; Yun et al., 2018; Shimizu et al., 2020). Some state-of-the-art methods involved in Raman analysis include confocal Raman microscopy, coherent anti-Stokes Raman scattering (CARS), stimulated Raman scattering (SRS), surface-enhanced Raman spectroscopy (SERS), and tip-enhanced Raman spectroscopy (TERS) (Malard et al., 2021; Itoh et al., 2023). Raman imaging is non-invasive, label-free, and requires minimal sample preparation (Ivleva, 2021). Once scanning of the sample surface to map the characteristic peak for imaging analysis is completed, it has the ability to visualize particles down to ∼100 nm (Sobhani et al., 2020). However, to achieve accurate imaging, several challenges need to be overcome. First, Raman imaging generates a large number of spectra that form a high-dimensional (hyperspectral) matrix (Liu et al., 2022). Interpreting and converting the matrix into a meaningful image is a complex step. The conversion process often requires the use of multivariate analysis for the extraction of key information for accurate mapping (Smith et al., 2019). Another challenge is the light diffraction issue. Diffraction of the Raman laser limits the lateral or spatial resolution of Raman images (e.g. ∼300 nm recommended by the instrument) and, thus, hinders the observation of small nanoplastics (Fang et al., 2020). To tackle this, image reconstruction can be carried out to collect useful signals while excluding unwanted noise.

The aim of this report is to monitor an undiscovered source of micro- and nanoplastics in daily life. Raman imaging was performed to capture and characterize the micro- and nanoplastics released in the kitchen, such as from the widely used silicone sealant around the sink area. To enable efficient imaging, several previously developed algorithms were refined to distinguish the silicone polymers from the coexisting additives (Fang et al., 2020; Luo et al., 2022b; Fang et al., 2022). An effort is also made to push the resolution limit of the confocal Raman microscope by performing image reconstruction, which benefits the detection of nanoplastics via deconvolution. The findings will potentially promote the progress of Raman imaging applications for micro- and nanoplastics’ research, and increase the need for comprehensive risk assessments of silicone products.

## 2 Materials and methods

### 2.1 Chemicals and samples

All chemicals including ethanol, acetone, and hydrogen peroxide (∼28%) were purchased from Sigma-Aldrich (Australia) and used as received. Milli-Q water (>18 MΩ cm) was used for the analysis. Silicones were purchased from a local market (Bunnings Warehouse, Australia) and are shown in Supplementary Figures S1–S3.

The real silicone samples were collected from a typical kitchen in Australia, as shown in Supplementary Figure S1, using a knife to scratch the sealing line. This approach aimed to mimic the cleaning and washing processes undertaken in everyday activities. Silicone has been applied for ∼7 years to seal a sink. The samples were cleaned using a mixture of ethanol and hydrogen peroxide (1:1, v/v) for 2 days at room temperature (∼24°C).

Beyond the real sample of the peeled or scratched debris from the sealant, the sealant residues attached to the kitchen items, such as the sink, bench, and tile, were also tested. Because it is difficult to directly test the sealant on the bench or tile, a method was applied to mimic the sealing application on a glass slide, as given in Supplementary Figure S2. The slides have been previously cleaned with ethanol, acetone, and Milli-Q water by sonication. Two glass slides are positioned to mimic an angle junction (corner) for sealing, one vertically and another horizontally. When applying/pressuring the silicone from the tube, the beginning part (∼10 cm) of the silicone tube was discarded to avoid possible contamination from the plastic tube and nose. Then, a wooden knife was employed to press and smoothen the silicone to the mimicked glass corner. Once dried after 24 h (as suggested by the introduction of the silicone product), the slides were tested after further washing with ethanol, to capture the residues or particles as the mimicked sample. Three brands of silicone (S#1–3) were tested here: a transparent one, a white one, and a gray one.

Another widely used application of silicone is for roof sealing. Similarly, the real sample from a metal roof where silicone has been applied for ∼7 years was also collected. The sealing process was mimicked on a glass slide, similar to that in the aforementioned kitchen, as shown in Supplementary Figure S3. Two brands (R#1–2) were tested, a white one and a gray one.

Different brands of silicone might generate varied results due to different formulations/ingredients/additives/colorants, working conditions (dry or wet), and configurations (applying skill, corner shape/material, etc.). Several typical brands were tested to obtain general information about the possibility of silicone releasing debris, whether in the context of kitchens or other uses, such as its use on roofs.

A scanning electron microscope (SEM) (Zeiss Sigma VP) was used to characterize the morphology of the microplastics and nanoplastics, in addition to energy-dispersive X-ray spectroscopy (EDS) detection. To this end, the sample was sputter-coated with a thin layer of platinum (∼6 nm) to increase the conductivity. The accelerated voltage was 10–20 kV with a working distance of 5–10 mm (Cowger et al., 2020).

### 2.2 Testing protocols and data treatment algorithms

The testing protocols follow previous reports (Sobhani et al., 2019; Luo et al., 2022b). In brief, Raman spectra were recorded using a WITec confocal Raman microscope (Alpha 300 RS, Germany) equipped with a 532-nm laser diode (<30 mW), under an objective lens (×100 or others) at room temperature.

To map the image, the laser was scanned on the sample surface to collect the signal at each pixel or point, as a scanning spectrum matrix, akin to a hyperspectral matrix. A previous report demonstrated the capability of Raman imaging to analyze polystyrene nanoplastics down to 100 nm (Sobhani et al., 2020). The methodology is validated herein and advanced with the following algorithms.

#### 2.2.1 PCA-based algorithm

The raw data from Raman scanning spectrum matrices were analyzed by PCA in OriginPro 2022 software, as reported previously (Luo et al., 2022b), to regenerate the PCA spectrum via the score (as the y-axis, with the wavenumber as the *x*-axis), and the PCA image via the loading coefficient (as the *z*-axis, with the scanning pixel position as the *x-/y-*axis). Depending on the presentation orientation of the raw data array, the score, and the loading coefficients can be swapped or transposed.

#### 2.2.2 Algebra-based algorithm

Two or more images, no matter the mapped Raman intensity or PC loading coefficients, can be merged using algebra functions, including “SUBTRACT” and “TIME,” in addition to using Origin software as well. For example, the loading coefficient was normalized to 0-1 (such as using function “(*x*_*i*_ *–x*_min_)*/*(*x*_max_ *–x*_min_)) first to avoid the bias from the weighting or the percentage of the eigenvalue variance (Luo et al., 2022b; Cheng et al., 2022). They can then be subtracted or multiplied with each other (as a merged one on the *z*-axis) to generate a “merged” image.

## 3 Results and discussion

### 3.1 SEM

**FIGURE 1:**
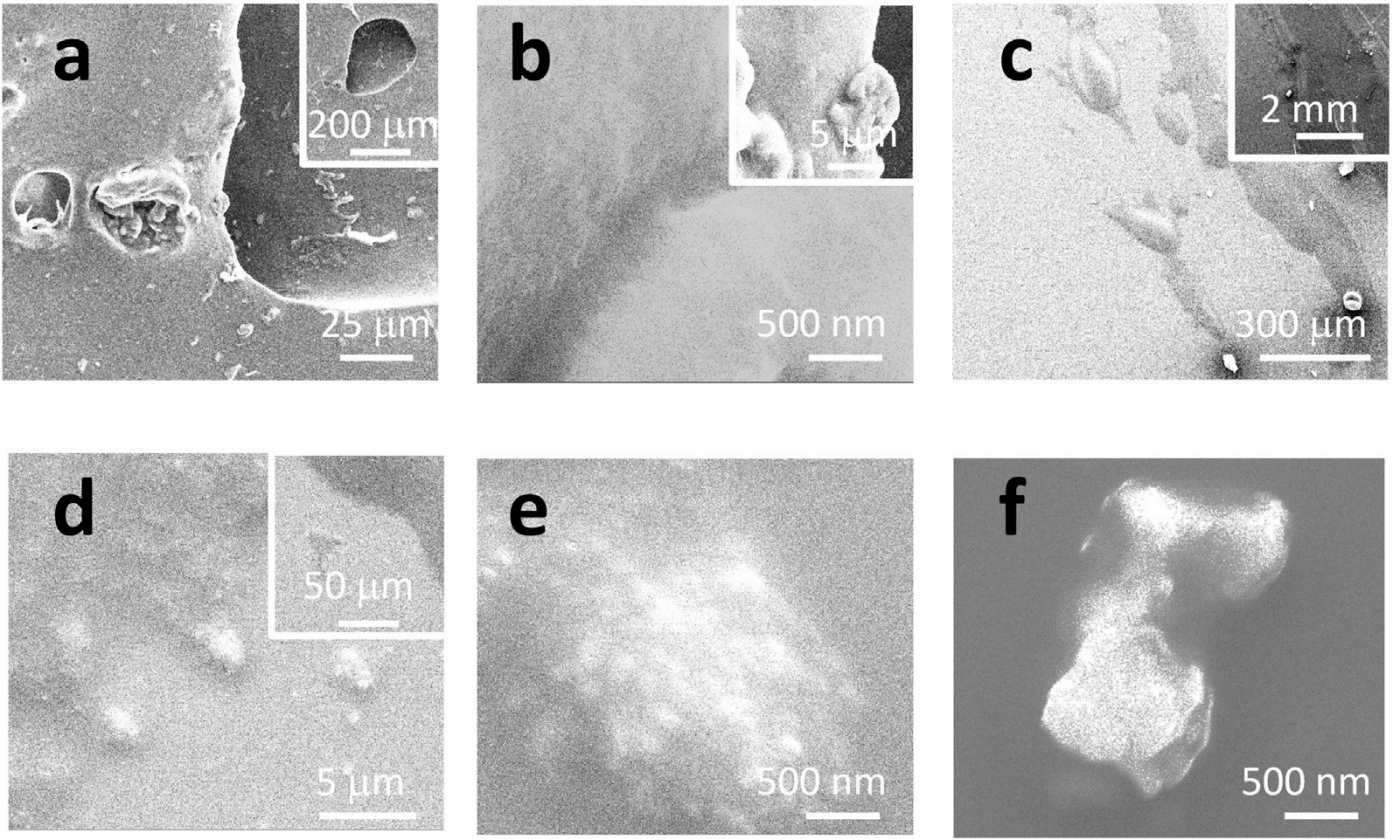
SEM images under different magnifications. **(A, B)** Real sample collected from a kitchen sink. **(C–F)** Mimicked sample on a glass surface.

Figure 1 shows the morphology of the sealant, including the real sample collected from a kitchen sink (a, b) and a mimicked sample (c–f) on a glass surface. For the real sample, some debris can be released, either due to the cleaning/washing that happens daily in the kitchen or aging and bio-degradation. In (a), the holes (from the dark/moldy area, as shown in Supplementary Figure S1) might be etched by bacteria, which is beyond the scope of this report. EDS can confirm that most of the debris is supposed to be silicone because of the appearance of peaks of C/O/Si, as shown in Supplementary Figure S4.

The sample was also prepared on a glass surface to mimic the sealing applications. The SEM image in Figure 1C suggests that the sealing line might be broken to release residues or debris, at microsize and nanosize, as detailed in (d–f). Some sub-structures are observed to have nanoparticles (likely the pigment or colorant) surrounded by a bulk like “glue or binder.” During the application process of silicone, the debris might originate either from the nose of the silicone tube or the pushing/pressing process by the wooden stick. The released debris can either stick to the tile/sink or be peeled off due to aging or cleaning. In the following sections, the debris will be tested to confirm whether or not they are made of silicone or silicone microplastics, or nanoplastics, depending on their sizes.

### 3.2 Silicone types

**FIGURE 2:**
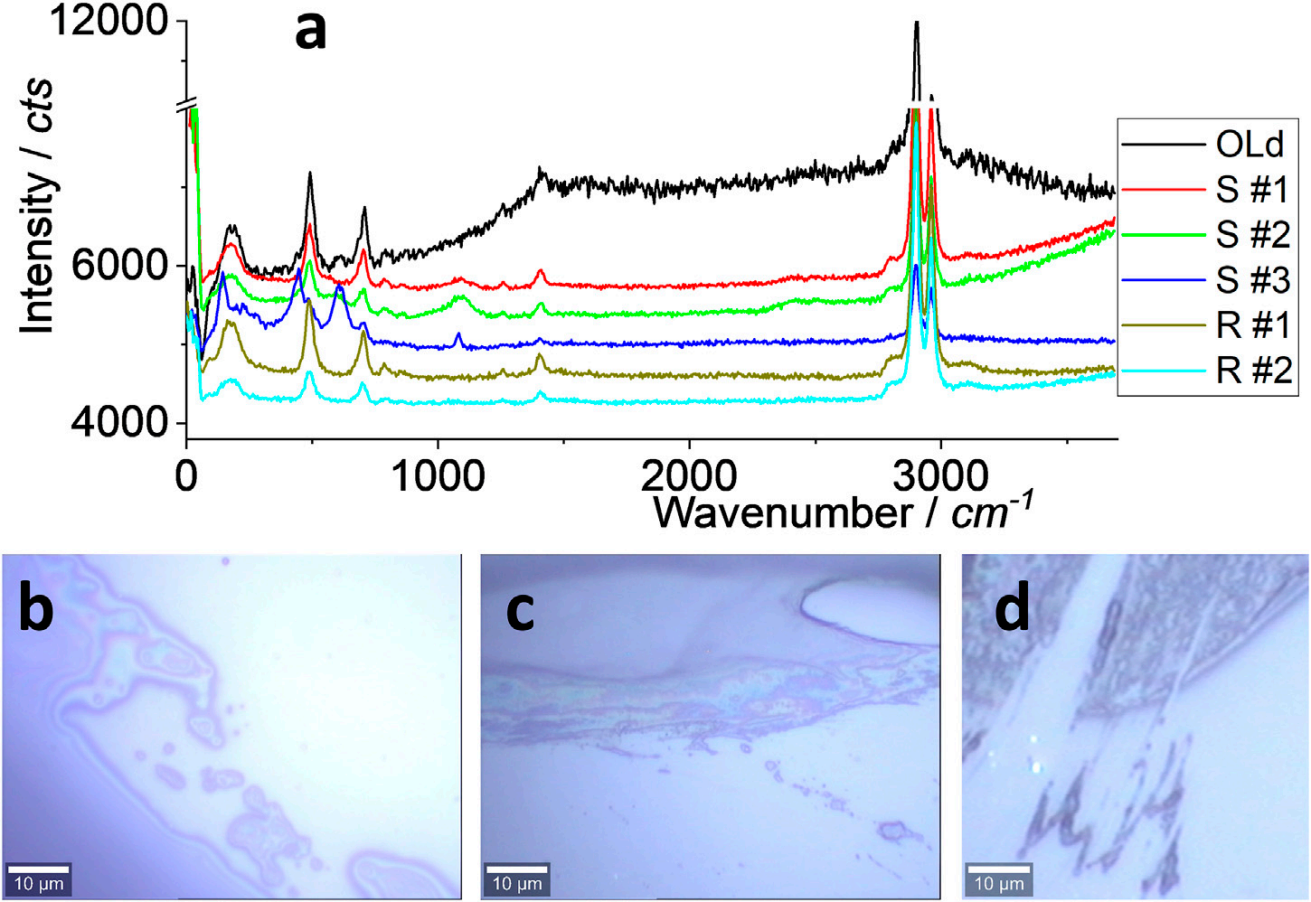
Raman spectra **(A)** and images **(B–D)**. All were collected under an objective lens of ×100, with an integration time of 10 s. The samples of “old” and “S#1–3” are for kitchen sealing, while “R#1–2” is for roof sealing. An “old” sample means that it was collected from a kitchen sealing after ∼7 years’ application (the real sample). The rest are newly prepared on a glass surface (the mimicked sample).

There are many types of silicone on the market. As said, three typical brands for kitchen application and two for roof sealing were selected. Although the ingredients might be different, the main component is silicone, such as 2-butanone, O, O’, O”–(methylsilylidyne) trioxime, as suggested by the brand tag. In this section, the Raman spectrum was used to identify the material, and the results are shown in Figure 2.

In Figure 2A, all spectra are similar, including the strong peak at ∼2,960 and ∼2,900 cm^−1^ assigned to C-H or N-H (Österle et al., 2015; Yun et al., 2018; Shimizu et al., 2020). The peaks at ∼1,410, ∼705, and ∼490 cm^−1^ are from Si-C and others such as colorants (Kassu et al., 2018). The peaks at ∼650 and ∼450 cm^−1^ for S#3 might be due to different additives or colorants. This test focuses on S#1 (transparent) and R#1 (white) as the model of silicone for kitchen and roof applications, respectively. In other words, their spectra are regarded as the “mother” to identify and assign the debris, either as microplastics or nanoplastics.

When silicone is applied in the kitchen or on the roof, there is a lot of debris, either along the sealing line, as shown in Figures 2B and C, or at the tip of the line, as shown in Figure 2D, both of which reflect the results shown in Figure 1. The released amount of debris might depend on the application skill, to-be-sealed materials, temperature, and other factors, which are discussed in the following sections. This report tests the possibility of silicone releasing debris as part of daily activities.

### 3.3 Real sample

This section focuses on the real sample of debris collected from 7-year-old silicone on a kitchen sink, as given in Supplementary Figure S1. After that, Figures 4–6 are analyzed for the mimicked sample of debris on the glass surface released from the fresh silicone, which are shown in Figures 2B–D/Supplementary Figure S2.

**FIGURE 3:**
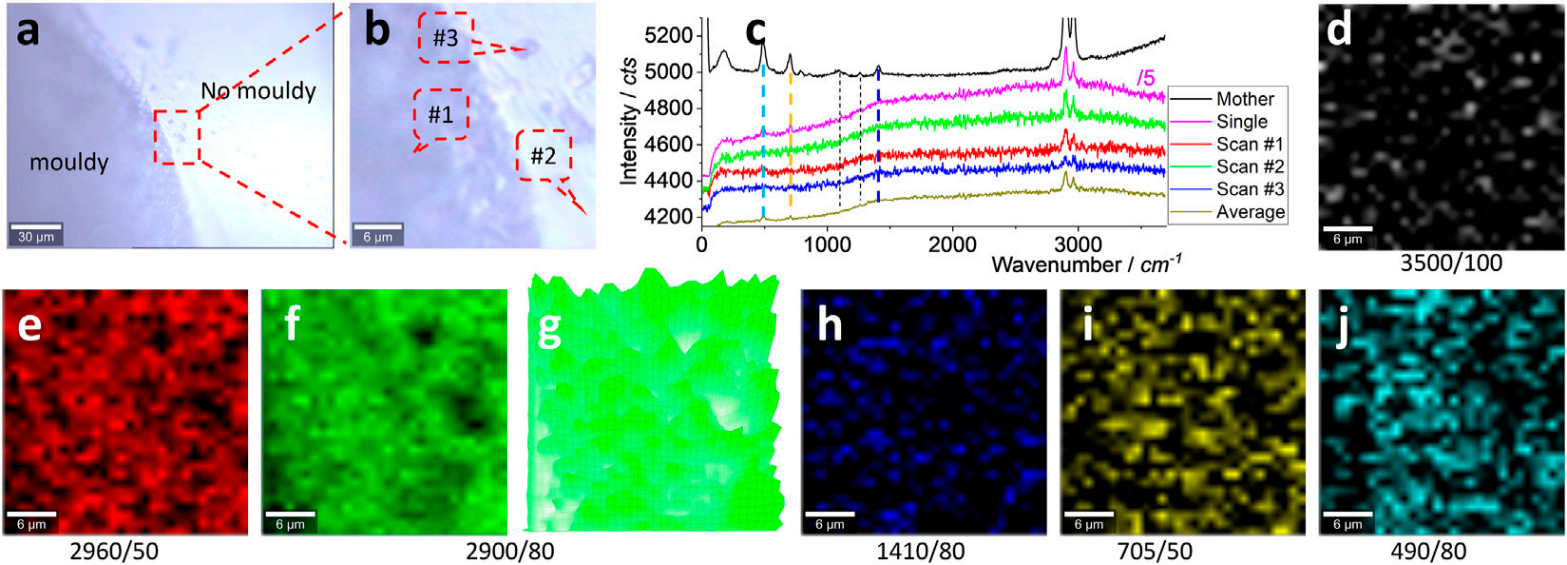
Images **(A, B)**, typical Raman spectra **(C)**, and Raman intensity images **(D–J)**. The squared area in **(A)** of 30 μm × 30 μm was zoomed in as **(B)** and scanned. Raman spectra were collected under an objective lens of ×100, with an integration time of 1 s for each pixel of 1 μm × 1 μm (to create a matrix of 30 × 30). **(C)** Raman spectra of the mother silicone, to compare with one single spectrum (10 s) and three typical scanning spectra (1 s) collected from the positions marked in **(B)**, and their average spectrum of 900 (30 × 30) spectra. The intensity images **(D–J)** are mapped at a blank wavenumber window **(D)**, the characteristic peaks of silicone **(E–J)**, as marked under each image (and the peak width), after 10% color off-setting. **(G)** is another version of **(F)**, using 3D presentation and a white background.

For the real debris sample in the kitchen collected from white silicone, some dark parts are noted as moldy, as shown in Figures 3A, B. It is noteworthy that there was no significant spectrum difference between the moldy and non-moldy areas, as shown in (c), from positions #1 and #2 marked in (b). These spectra were collected during the scanning process, with an integration time of 1 s, including another position (#3). All the characteristic peaks shown in Figure 2 are marked with dashed lines (some clear and some blurred). If the integration time is prolonged from 1 to 10 s, a single spectrum was collected as well and the clear characteristic peaks are better than the mother spectrum. The strong peaks at ∼2,960 and ∼2,900 cm^−1^ can be attributed to silicone debris.

The scanning spectrum matrix was mapped to visualize the silicone and to increase the sensitivity, from a statistical point of view. In other words, the scanning spectrum matrix contains 900 (30 × 30) spectra and can generate an image with a chance to average the background noise, as reported previously (Luo et al., 2022b; Cheng et al., 2022). In Figure 3C, the averaged spectrum can present the main peaks at ∼2,960, ∼2,900, ∼1,410, ∼705, and ∼490 cm^−1^, supporting the aforementioned assumption.

Before mapping the characteristic peaks of silicone, a blank wavenumber window where the silicone has no signal, 3,450–3,550 cm^−1^, is used to generate an image shown in Figure 3D as an internal reference of the image background. Only random noise is mapped. On the contrary, the characteristic peaks of silicone can generate images (e–j), and all of them (except (h), due to its intrinsically weak peak) are different from (d), suggesting the presence of silicone. Among them, the strong peaks at ∼2,960 and ∼2,900 cm^−1^ can map the clear pattern. The remaining weak peaks generate blurred patterns in (h–j), particularly (i, j) still different from (d), such as in the middle parts. (g) is another version of (f), generated using 3D presentation. This strong peak’s mapping image will be selected to visualize the silicone in the following parts.

There is no difference between the moldy and non-moldy parts, meaning the silicone is still dominating the Raman scattering. Perhaps the sample preparation has removed the moldy signal, or the moldy signal has not been effectively picked up (being masked and dominated by the silicone signal). Meanwhile, as reported, the sink, bench, or tiles in a kitchen cannot be taken directly to the laboratory for tests in order to capture the debris. The glass slide was instead used to mimic the sealing in a kitchen, and taken to the laboratory for tests, as shown in the following sections.

### 3.4 Mimicked sample

#### 3.4.1 Microplastics

**FIGURE 4:**
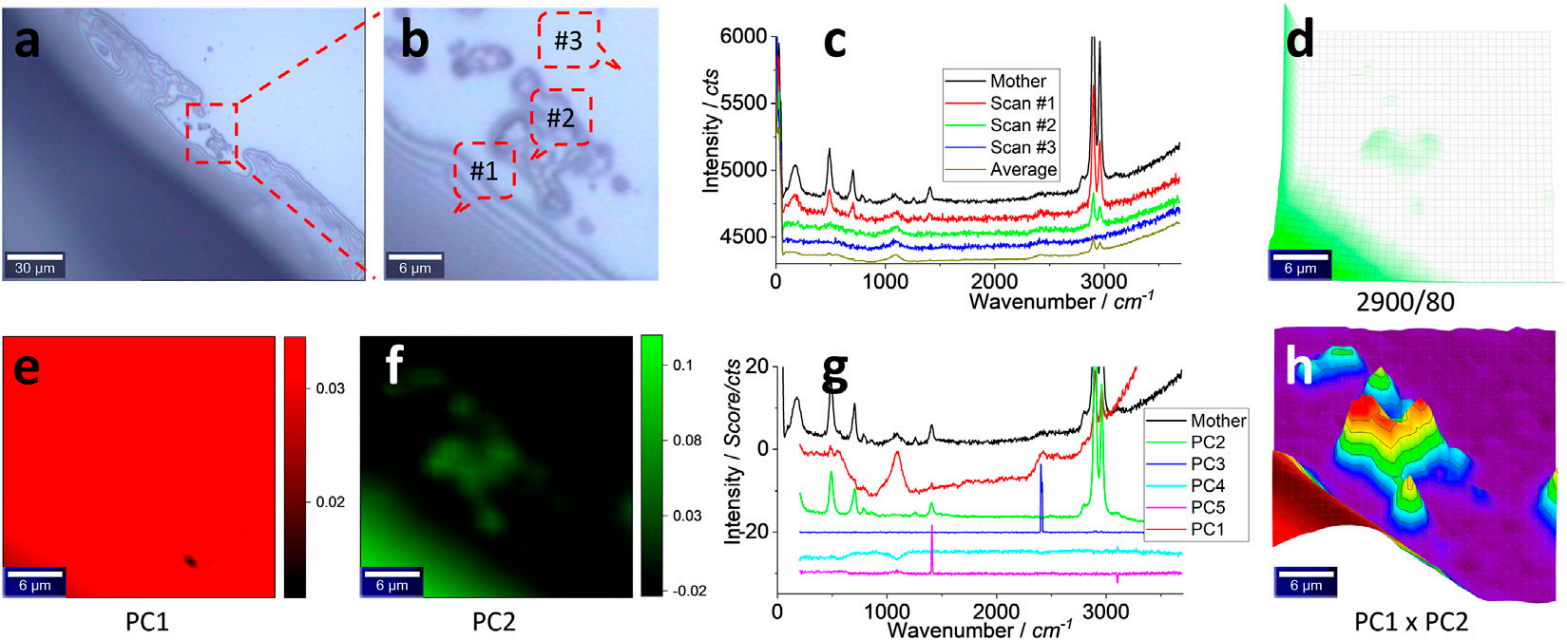
Images **(A, B)**, typical Raman spectra **(C)** and image **(D)**, PCA images of PC1 **(E)** and PC2 **(F)**, PCA spectra **(G)**, and PCA image merging of PC1 and PC2 **(H)**. The squared area in **(A)** of 30 μm × 30 μm was zoomed in as **(B)** and scanned. Raman spectra were collected under an objective lens of ×100, with an integration time of 1 s for each pixel of 1 μm × 1 μm (to create a matrix of 30 × 30). **(C)** Raman spectra of the mother silicone, to compare with three typical scanning spectra (1 s) collected from the marked positions in **(B)** and their average spectrum of 900 (30 × 30) spectra. The intensity image **(D)** is mapped at a characteristic peak of silicone at 2,900 cm^−1^, after 10% color off-setting. After PCA, the loading coefficients of PC1–PC2 are mapped as images **(E, F)**. **(G)** PCA spectra, with the mother spectrum as the reference. **(H)** merges **(E, F)**, using 3D presentation and a white background, after normalizing the loading coefficients to 0–1.

The results of the mimicked sample on the glass surface are presented in this section, using silicone S#1 as a model (Supplementary Figure S2). The image in Figures 4A, B shows the interference of the illumination light, which suggests the thin layer of silicone. Once zoomed in as (b) and scanned, the typical spectra from the marked positions are shown in (c), along with the average spectrum from the 900 spectra in the scanning spectrum matrix. When compared with the mother spectrum, it can again be assigned to silicone for Scans #1 and #2. Scan #3 can show the background spectrum that is assigned to glass. The averaged spectrum yields strong peaks at∼ 2,900 and ∼2,960 cm^−1^, suspected to be silicone.

Again, the scanning matrix is mapped to generate an image shown in Figure 4(d), from the strongest peak at ∼2,900 cm^−1^ (the remaining peaks’ images are given in Supplementary Figure S5). The image matches well with that in (b), confirming the presence of silicone.

However, the image in (d) does not present all particles in (b). Perhaps the strong signal from the left-bottom part, the bulk silicone layer (#1 in b), shields the weak signal from the particles in the central part. Imaging sensitivity can be increased to capture them, using chemometrics.

In other words, the image shown in Figure 4D is mapped from a solo peak at ∼2,900 cm^−1^. The remaining signal in the spectrum is ignored. Chemometrics, such as PCA, can be used to decode the scanning matrix more efficiently, to map silicone from the whole set of the spectrum, rather than from the solo peak only, to increase the sensitivity (Luo et al., 2022b; Cheng et al., 2022). PCA can ideally decompose the spectrum matrix into two new matrices, one containing the spectrum profile to identify the item by comparing with the mother or standard spectrum and another containing the intensity information for mapping. The results are given in the bottom row of Figure 4.

The images of PC1 and PC2 are shown in Figures 4E and F, respectively, by mapping their loading coefficients (as PCA intensity). Although (e) looks like the image background, (f) matches well with (b) and maps more particles than (d), confirming the aforementioned assumption of PCA to increase the sensitivity. The PCA spectrum in (g) can support the assignment because the PC1 spectrum has a non-flat baseline that looks similar to Scan #3 in (c), and it is assigned to the background. However, the strong peaks at ∼2,960 and ∼2,900 cm^−1^ mean that the contribution from silicone should also be taken into account. On the contrary, the PC2 spectrum looks similar to the mother spectrum and is dominated by silicone. The rest are assigned to the noise or the PCA calculation variation. More PCA parameters are given in Supplementary Figure S6).

However, PCA is not a supervised analysis. From the PCA results, the signal can be further treated toward enhancement. For example, since PC1 contains some information on silicone, even being dominated by the background, it can be merged with PC2 to further extract silicone information (Luo et al., 2022b; Cheng et al., 2022). To this end, there are many functions that can be employed, but a time/multiply algebra one was selected. The result is shown in Figure 4H to better present the silicone particles in the central part.

In other words, the loading coefficients were first normalized to (0, 1) in order to avoid bias. They were then multiplied as a merged one to generate the image (h). After being normalized to a range of 0–1 and time together, the stronger contributions (of the PCA intensity) from PC1 and PC2 can be simultaneously picked up and mapped in the merged one. Consequently, the silicone microplastics in the central part are captured.

#### 3.4.2 Nanoplastics

**FIGURE 5:**
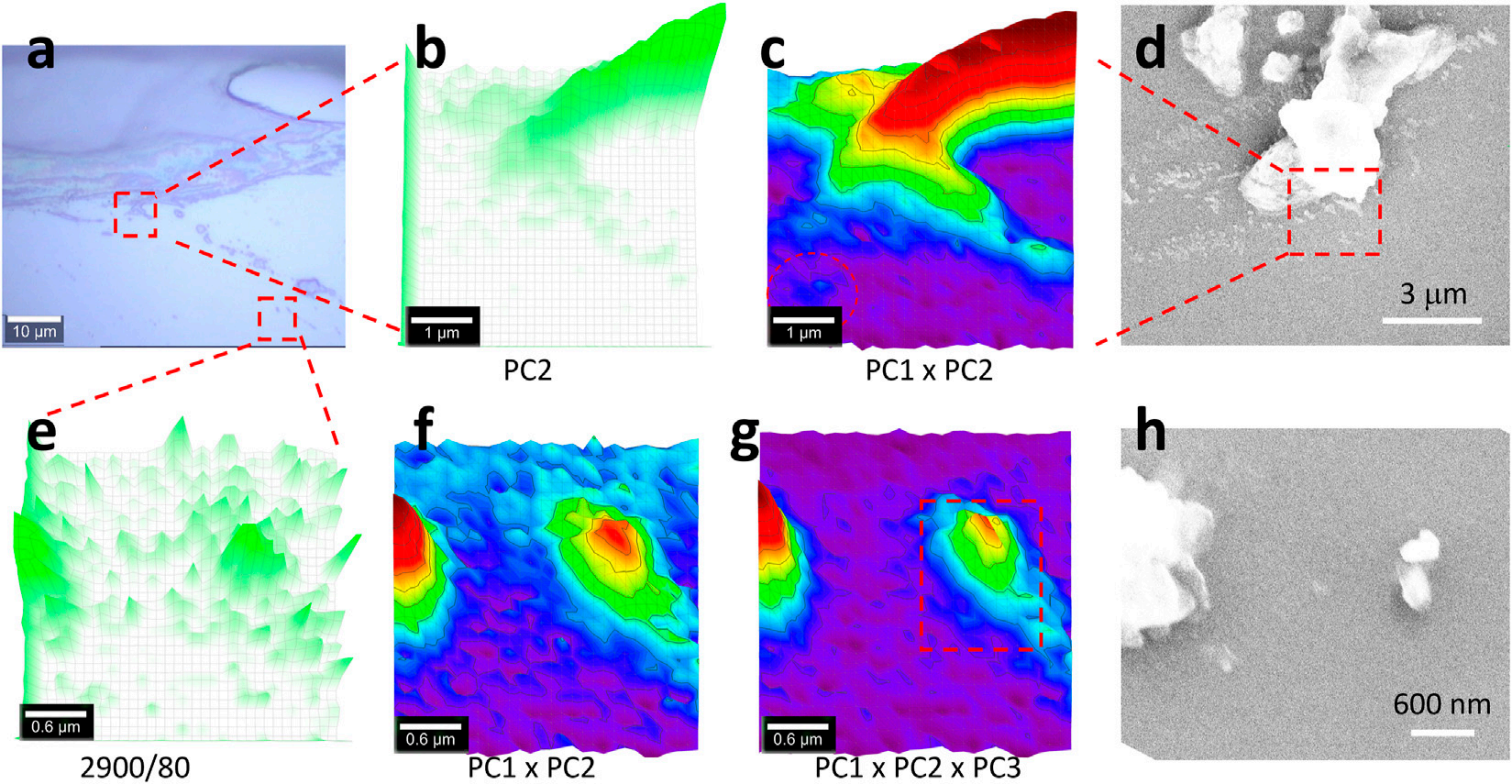
Image **(A)**, PCA images **(B,C,F, G)**, Raman image **(E)**, and SEM images **(D, H)**. The central-squared area in **(A)** of 5 μm × 5 μm was scanned as **(B–D)**, while the bottom-right squared area of 3 μm × 3 μm was scanned as **(F–H)**. Raman spectra were collected under an objective lens of ×100, with an integration time of 1 s for each pixel of 0.17 μm × 0.17 μm **(B, C)**, or 0.1 μm × 0.1 μm **(E–G)** (to create a matrix of 30 × 30 in both cases). **(E)** maps the Raman intensity at 2,900 cm^−1^ as a reference. After the PCA, the image **(B)** maps PC2’s loading coefficient, while **(C, G, G)** merge the different PCs’ loading coefficients after being normalized to 0–1, using 3D presentation and a white background. **(D, H)** SEM images at the corresponding positions. The squared area in **(G)** is further analyzed in Figure 6.

In this section, the scan is further zoomed in, in order to capture nanoplastics. The testing position was also changed, as shown in Figures 4, 5, to show more testing results in random positions. In Figure 5, the sealing line boundary also releases debris. In (a), the two squared areas are tested individually and presented on the top and bottom rows, respectively.

Due to the high sensitivity, as shown in Figure 4, herein, only the PCA results are shown, including the PC2 image in Figure 5B and the merged image in (c). More results are given in Supplementary Figures S7–S9. The image in (b) has been improved in (c) due to the extra contribution from PC1, similar to that in Figure 4. In the left bottom part, a nanoplastic is patterned and circulated in (c) but not in (b), suggesting improvement. However, when compared with the SEM image in (d), some details are missed. As reported, confocal Raman imaging can effectively pick up the signal from the focal plane. In the off-focal plane along the *z-*axis, the signal cannot be effectively picked up. Furthermore, for Raman imaging, the generated image along the *z-*axis is the intensity value, rather than the physical height (Sobhani et al., 2019).

It should be noted herein that in this study the scanning pixel size was 0.17 × 0.17 μm, which is much smaller than the recommended scanning resolution that is defined by the full width at half-maximum of a Gaussian peak (FWHM, *0.51λ/NA* or *λ/2NA*, ∼300 nm if taking wavelength *λ* of the laser as 532 nm and the numerical aperture *NA* of 0.9) because the laser spot power density is axially transcended and follows a Gaussian distribution. The reason for us reducing the scanning pixel to smaller than the laser spot is because the central part of the laser spot (centroid), or the summit of the Gaussian peak, has the strongest power density to emit the strongest Raman signal (Fang et al., 2020). In this case, there is a better chance to capture small particles, once the nanoplastic is positioned at the center of the laser spot to emit the signal stronger than when being positioned off-center. In other words, to better position the nanoplastic at the center of the laser spot, a reduction in scanning pixels is needed, as conducted here.

Another testing area shown in Figure 5A is presented in (e–h), with the Raman image mapping at 2,900 cm^−1^ in (e) as a reference. It can be seen that many particles are patterned but might originate from noise or signal variation. Again, PCA was employed to enhance the sensitivity. Different from the aforementioned situation, herein, the PC3’s spectrum also contains the contribution from silicone, due to the appearance of strong peaks at 2,900 and 2,960 cm^−1^. Consequently, the merged (PC1 and PC3) image in (f) can be further improved in (g), by picking up the extra contribution from PC3. This algebra-based algorithm can intentionally correct the non-supervised PCA and is thus recommended for microplastics analysis, particularly for nanoplastics that emit a weak Raman signal (Luo et al., 2022b; Cheng et al., 2022).

In brief, two nanoplastics were patterned, as shown in Figures 5F, G, by shrinking the scanning pixel size from 1 μm × 1 μm in Figures 3, 4 to 0.17 × 0.17 μm in Figures 5B, C and 0.1 × 0.1 μm in (e–g). We further zoomed in on the squared area in (g), and the results are shown as follows. The SEM image in (h) looks slightly different from (g) again, due to the difference between confocal Raman imaging and the SEM image, as discussed previously. Not every particle in the SEM image is mapped in the Raman image, either due to the Raman image resolution limit or the weak/no Raman signal picked up.

#### 3.4.3 High resolution and image reconstruction

**FIGURE 6:**
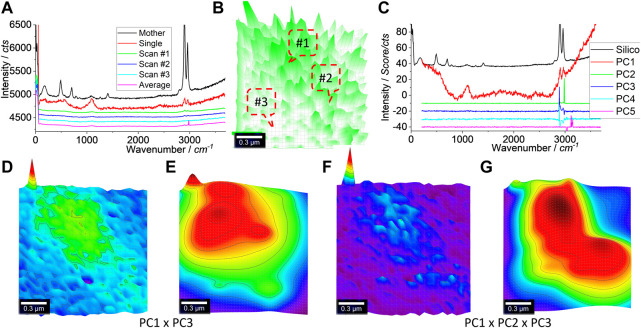
Raman spectra **(A)** and image **(B)**, PCA spectra **(C)**, and images **(D, F)** toward image reconstruction **(E, G)**, respectively. An area of 1.5 μm × 1.5 μm was scanned, as squared in Figure 5G. Raman spectra were collected under an objective lens of ×100, with an integration time of 1 s for each pixel of 50 nm × 50 nm (to create a matrix of 30 × 30). After the PCA, the Raman spectrum of the mother silicone is shown in **(A, C)** as a reference. **(D, F)** merge the different PCs’ loading coefficients after being normalized to 0–1, as suggested. **(E, G)** are the reconstructed versions of **(D, F)**, by 2D Gaussian surface fitting.

We further zoomed in on the scan area squared in Figure 5G, and the generated results are shown in Figure 6. In (a), the typical Raman spectra are shown. Just like before, three typical spectra including a blank curve are presented. Only the strong peaks at 2,900 and 2,960 cm^−1^ can be identified. The mapped image is shown in (b). In the central part, the intensity is higher than in other areas and, thus, patterned.

PCA was similarly employed to increase the mapping sensitivity and certainty. The PCA spectra are shown in Figure 6C. Again, PC1 is dominated by the background (the glass’s Raman scattering) along with the contribution from silicone, due to the appearance of peaks at 2,900^–1^ and 2,960 cm^−1^. PC2 and PC3 also share these two peaks, but PC2 appears to contain some noise, as discussed in Supplementary Figure S10.

Using the algebra function again, they are merged, as shown in Figures 6D and F, either by PC1 × PC3 (d) or by PC1 × PC2 × PC3 (f). Both can pattern the central part to match well with the squared area, as shown in Figure 5G. Due to the decreased pixel size (50 nm × 50 nm), the laser spot might cover a much bigger area (∼720 nm; *1.22λ/NA*) than the pixel size, which leads to the blurred pattern here. In order to overcome this issue, the image can be reconstructed by fitting with a Gaussian surface. The results are shown in (e, g). (g) looks like there are two particles aggregated together, reflecting the SEM image shown in Figure 5H.

The reason for the image reconstruction using a Gaussian surface is because the laser spot behaves as an axial transcended distribution. Due to diffraction, the power density within this laser spot follows a Gaussian distribution in 2D or a Gaussian surface. Thus, it is supposed that the emitted Raman scattering also follows a Gaussian surface, in terms of Raman intensity (or PCA intensity) (Fang et al., 2020). The Gaussian fitting (2D) was thus used to reconstruct the images as (e, g) toward deconvolution. Better presentations are achieved but more research is needed to, for example, identify whether the pattern is an individual particle or an aggregate of several particles, as shown in Figure 5G. The fitting parameters are given in Supplementary Figure S11 and Supplementary Tables S1, S2.

### 3.5 Particle analysis

Silicone for roof sealing, including the real sample, was also tested, along with the mimicked sample on the glass surface again (Supplementary Figures S12, S13). Basically, similar results are found: silicones can release microplastics or nanoplastics in potential. The outdoor release might generate a different concern from the indoor release (around the sink where to prepare food and clean cooking wares), but once they enter the environment, they might still pose risks eventually. The risk assessment is, thus, urgently needed.

The release amount can also be estimated. From the images shown in Figure 2, it was estimated that 2–10 debris/10 μm is released along the sealing boundary. A sink in the kitchen usually has a size of 0.5–1.5 m, which means 0.1–1.5 million for one-side sealing, or 0.4–6 million debris for four-side sealing, in potential. These individual debris pieces, no matter if they are microplastics or nanoplastics, might be peeled off and contaminate the food or enter the environment via the sink. Currently, it is difficult to estimate the once-release amount (such as being peeled off, during one washing/cleaning process) or chronic-release amount during the whole lifetime of the sealant. The test here also suggests that nanoplastics of silicone can be released, which might be an even bigger concern, given the toxicity has not yet been addressed, and might be more serious than microplastics (Editorial, 2019; Sun et al., 2020; Ivleva, 2021).

## 4 Conclusion

This study demonstrates that Raman imaging can effectively characterize microplastics and particularly nanoplastics, even smaller than the recommend scanning resolution of the confocal Raman imaging that is defined by the diffraction of the laser. While efforts have been made to push the limit toward the breakthrough of the diffraction limit, the signal should be well extracted to avoid the false positive/negative characterization of microplastics and particularly nanoplastics, which generally emit weak signals.

This study assesses the silicone sealants that are widely used in kitchens. As a case study, the results endorse the possibility that the silicone sealant can release microplastics and nanoplastics, meaning a risk assessment should be conducted. Beyond the emerging contamination concerns relating to particulate matters themselves, silicone microplastics and nanoplastics might also contain harmful additives, such as phthalates which are known endocrine disruptors. People should be encouraged to prepare food in areas where food will not come into direct contact with the sealant and clean the areas in the vicinity of the sealant regularly to remove detached silicone fragments.

## Data Availability

The original contributions presented in the study are included in the article/Supplementary Material; further inquiries can be directed to the corresponding authors.
