# Supplementary material for: Raman imaging for the analysis of silicone microplastics and nanoplastics released from a kitchen sealant
Source: Front Chem. 2023 May 17;11:1165523. doi: 10.3389/fchem.2023.1165523 (PMC10229840; doi:10.3389/fchem.2023.1165523)
Supplement: Supplementary file 1 [file DataSheet1.docx]

Supplementary Material

Raman imaging for the analysis of silicone microplastics and nanoplastics released from kitchen sealant

Cheng Fang*, Yunlong Luo, Ravi Naidu

*** Correspondence:** Corresponding Author: [cheng.fang@newcastle.edu.au](mailto:cheng.fang@newcastle.edu.au)

Contents

[1 Figures S1-S3: photo images 3](#_Toc132744822)

[2 Figure S4: EDS 5](#_Toc132744823)

[3 Figure S5: more Raman images for Figure 4 6](#_Toc132744824)

[4 Figure S6: more PCA parameters for Figure 4 6](#_Toc132744825)

[5 Figure S7: more Raman images for Figure 5 7](#_Toc132744826)

[6 Figures S8-S9: more PCA analysis parameters for Figure 5 9](#_Toc132744827)

[7 Figure S10: more PCA analysis parameters for Figure 6 11](#_Toc132744828)

[8 Figure S11 / Tables S1-S2: more fitting analysis parameters for Figure 6 12](#_Toc132744829)

[9 Figure S12: real roof sample 19](#_Toc132744830)

[10 Figure S13: mimicked roof sample 20](#_Toc132744831)

# Figures S1-S3: photo images


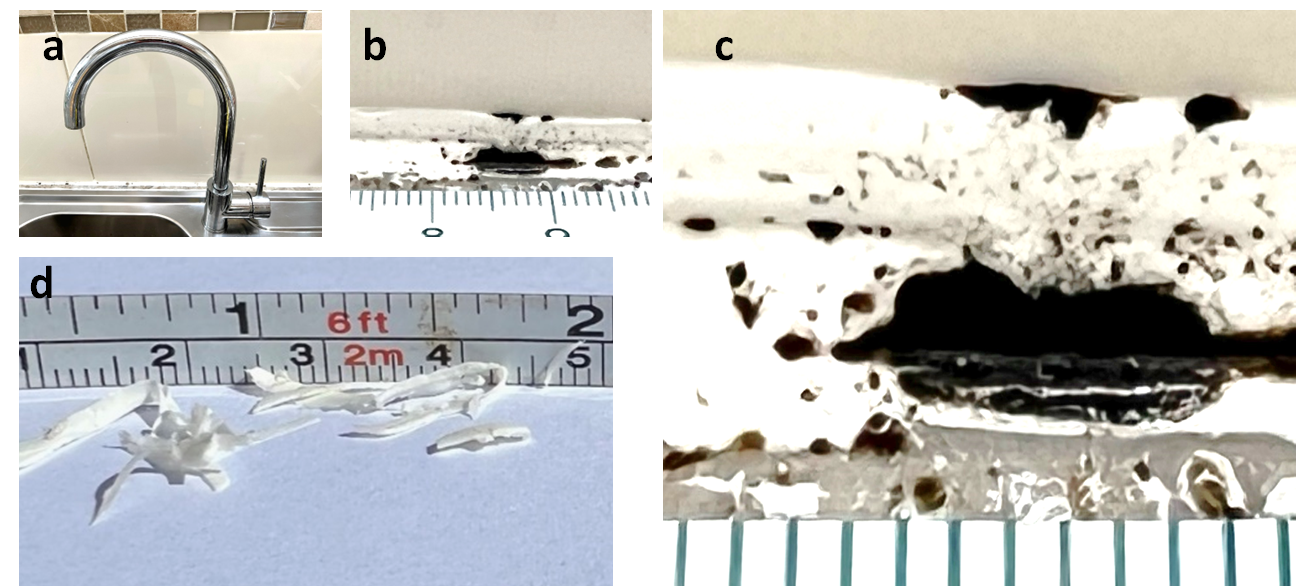


**Figure S1**. Photo images of silicone around a sink in a kitchen. (a) is zoomed in as (b) and (c), to show the mouldy of the silicone sealant (dark parts). (d) shows the collected samples for test. The mini scale in the ruler is 1 mm.

Figure S1 shows the silicone in a kitchen, applied ~7 years ago. The mouldy part can be seen as dark dot and has been collected for test. However, after cleaning, they look white in (d). While some debris are bigger than 5 mm, some are also smaller than 5 mm (particularly in (c)) that can be categorised as microplastics in potential.


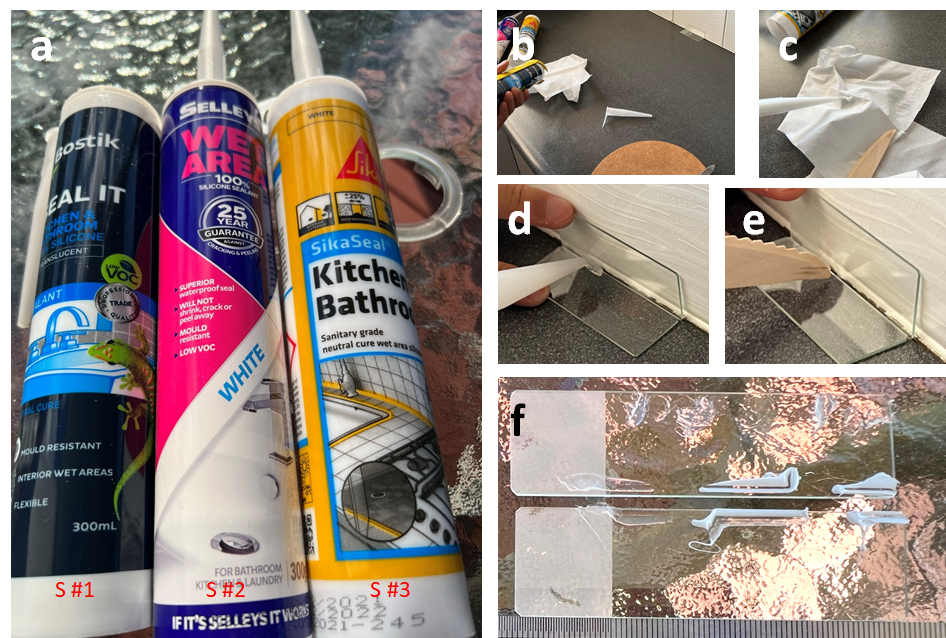


**Figure S2**. Photo images of silicones and the mimicked sealing. (a) shows 3 brands of silicone. (b) demonstrates how to apply the silicone on the glass slides in a kithen. (c) is to remove the first part of silicone in the tube to avoid the possible cross-contamination. (d, e) show the applying process. (f) is the samples for test.

We mimic the sealing process in kitchen, on glass surface, as demonstrated here. Three silicones are tested in (a). The sealing process is demonstrated in (b-e). After drying, the samples in (f) are ready for test.


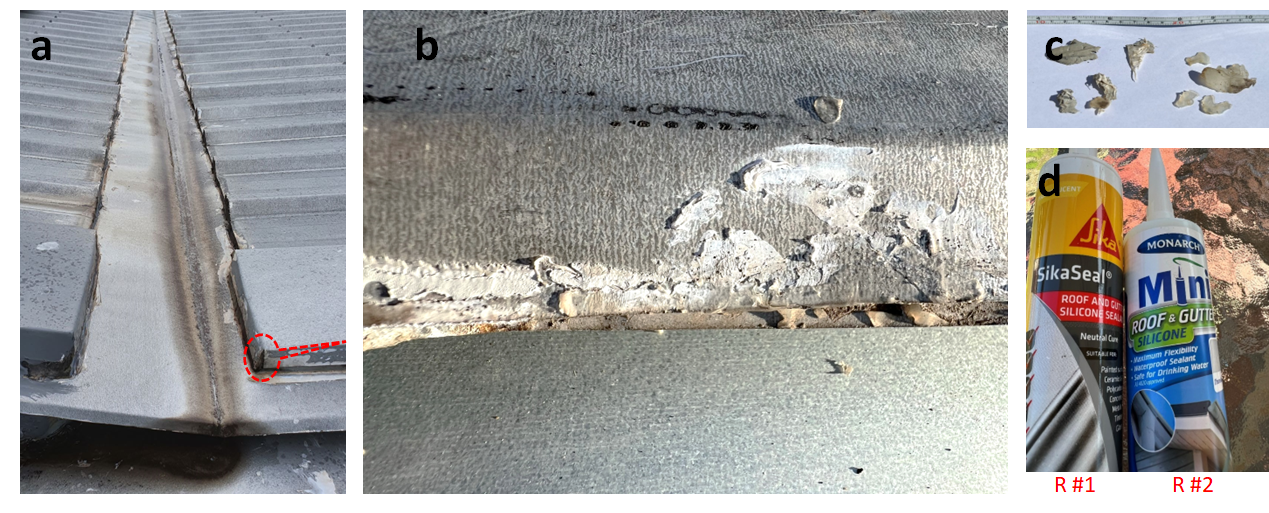


**Figure S3**. Photo images of the roof sample. (a) is the metal roof, the corner was sealed by silicone ~7 years ago and zoomed in as (b). (c) is the real samples collected directly from the metal roof and (d) shows the fresh silicones for test.

The roof samples are shown here. The test is for comparison with the kitchen samples.

# Figure S4: EDS


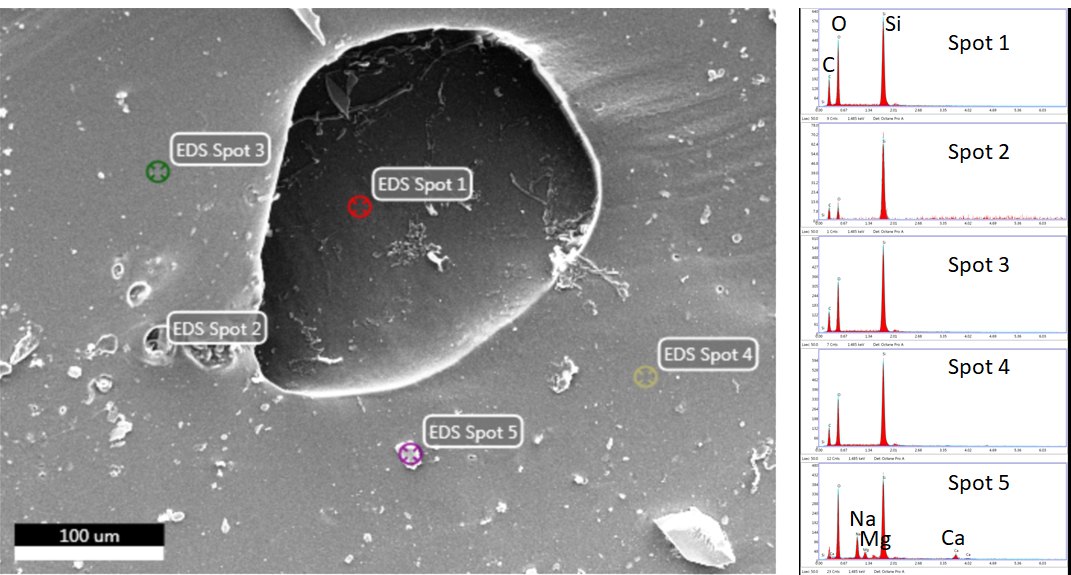


**Figure S4**. EDS and SEM collected from the real sample shown in Figures 1(a, b).

The EDS are shown here, which are collected from the marked position in the SEM image. In the mouldy area (etched as holes) in Spot 1-2, the EDS is similar with these from the non-mouldy area in Spot 3-4. All of them are dominated by C/O/Si, or silicone. Spot 5 might be a mineral dust.

# Figure S5: more Raman images for Figure 4


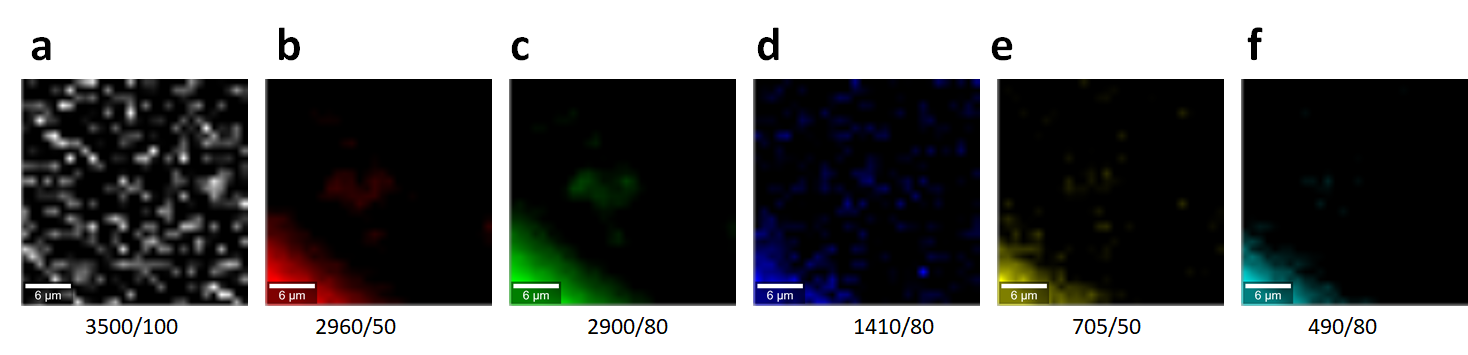


**Figure S5**. Raman images for Figure 4, including the internal reference (a) and the characteristic peaks’ images (b-f).

The images here basically present the characteristic peaks’ patterns, to confirm the assignment of silicone in the scanning area.

# Figure S6: more PCA parameters for Figure 4


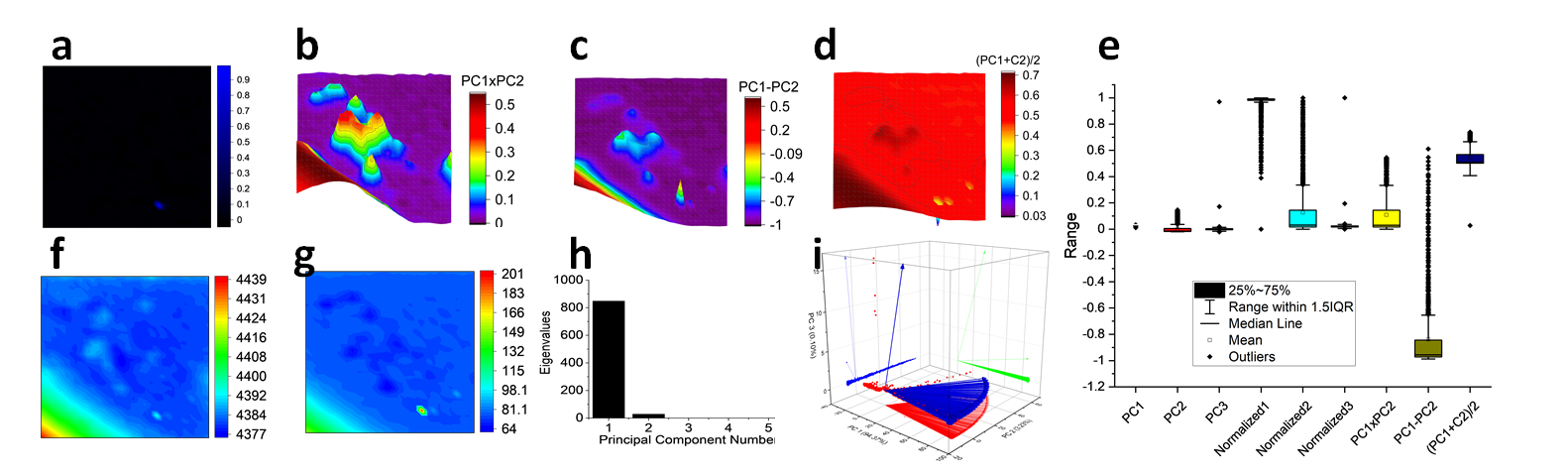


**Figure S6**. PCA analysis parameter for Figure 4. (a-d) are the images mapping the loading coefficients of PC3 (a) and the merged PC1 and PC2 (b-d), using different algebra functions, as indicated in the colour scale bars. (e) shows the distribution ranges of the loading coefficients, including before and after normalisation, and being subjected to merge. (f) is the mapped mean, while (d) the standard deviation of the PCA calculation. (h) is the scree plot and (i) the bi-plot.

Figure S6(a) shows only one dot or pixel pattern for PC3, which might be due to the cosmic ray. It does thus not take part in the algebra calculation to extract the silicone information. The different extraction using the algebra functions generate the images (b-d). The loading coefficients can be compared in (e). (f) is the mapped mean to visualise the area where the main signals or variance are collected. (g) implies the areas where we should pay more attention to the analysis results. (h) shows the main variance of the eigenvalues has been taken by PC1. (i) suggests the main PCs, including PC1-PC3, are almost independent from each other, which is the nature of the PCA analysis.

# Figure S7: more Raman images for Figure 5


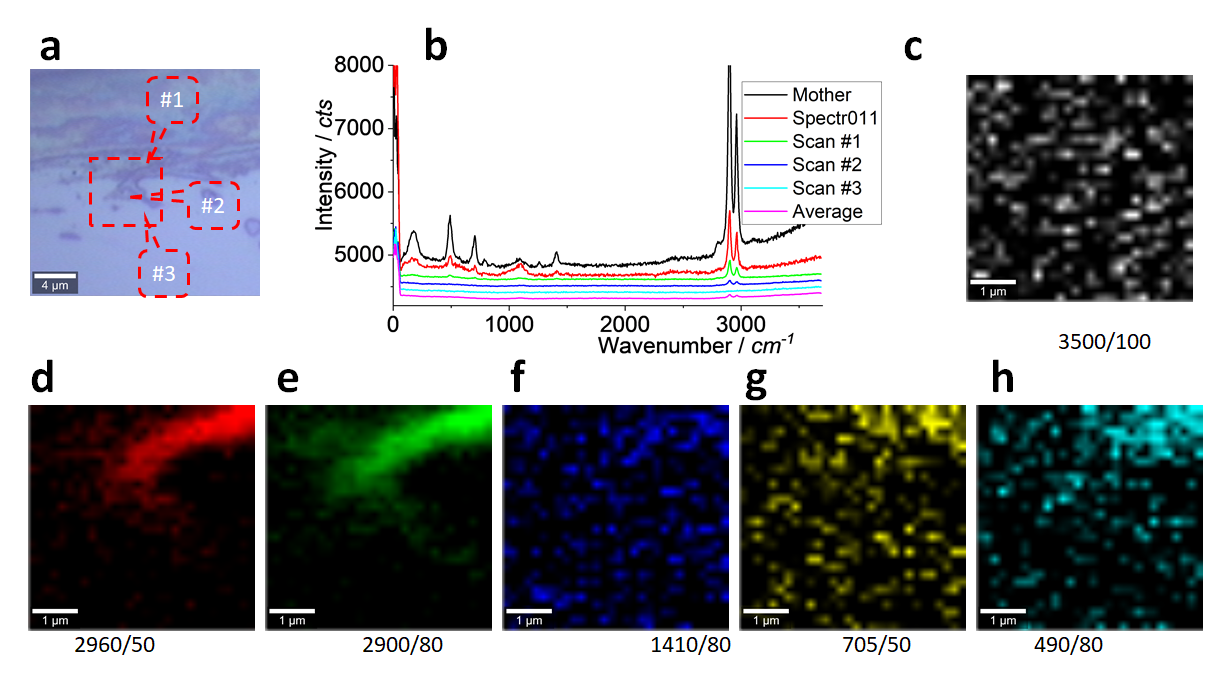


**Figure S7**. More Raman images for Figure 5. (a) is the photo image, with marks to suggest the testing positions. (b) is the Raman spectra. (c) maps the blanks as a reference image, (d-h) map the characteristic peaks of silicone, after 10 % colour off-setting. The squared area in (a) of 5 μm × 5 μm was scanned. Raman spectra were collected under an objective lens of 100×, integration time of 1 s for each pixel of 0.17 μm × 0.17 μm (to create a matrix of 30 × 30). (b) shows the Raman spectra of the mother silicone, to compare with 3 typical scanning spectra (1 s) collected from the marked positions in (b), and their average spectrum of 900 (30 × 30) spectra.

Herein more information about the Raman imaging in Figure 5 is provided. The photo image in (a) marks the testing positions for scan and for signal collection. During the scanning process, the typical Raman spectra we collected are shown in (b). A single spectrum (#011) is also shown for comparison, due to the longer integration time (10 s vs. 1 s). (c) is an internal reference image to suggest the background. (d-h) map the characteristic peaks of silicone again. Basically, we can confirm the presence of silicone, from the patterned area.

# Figures S8-S9: more PCA analysis parameters for Figure 5


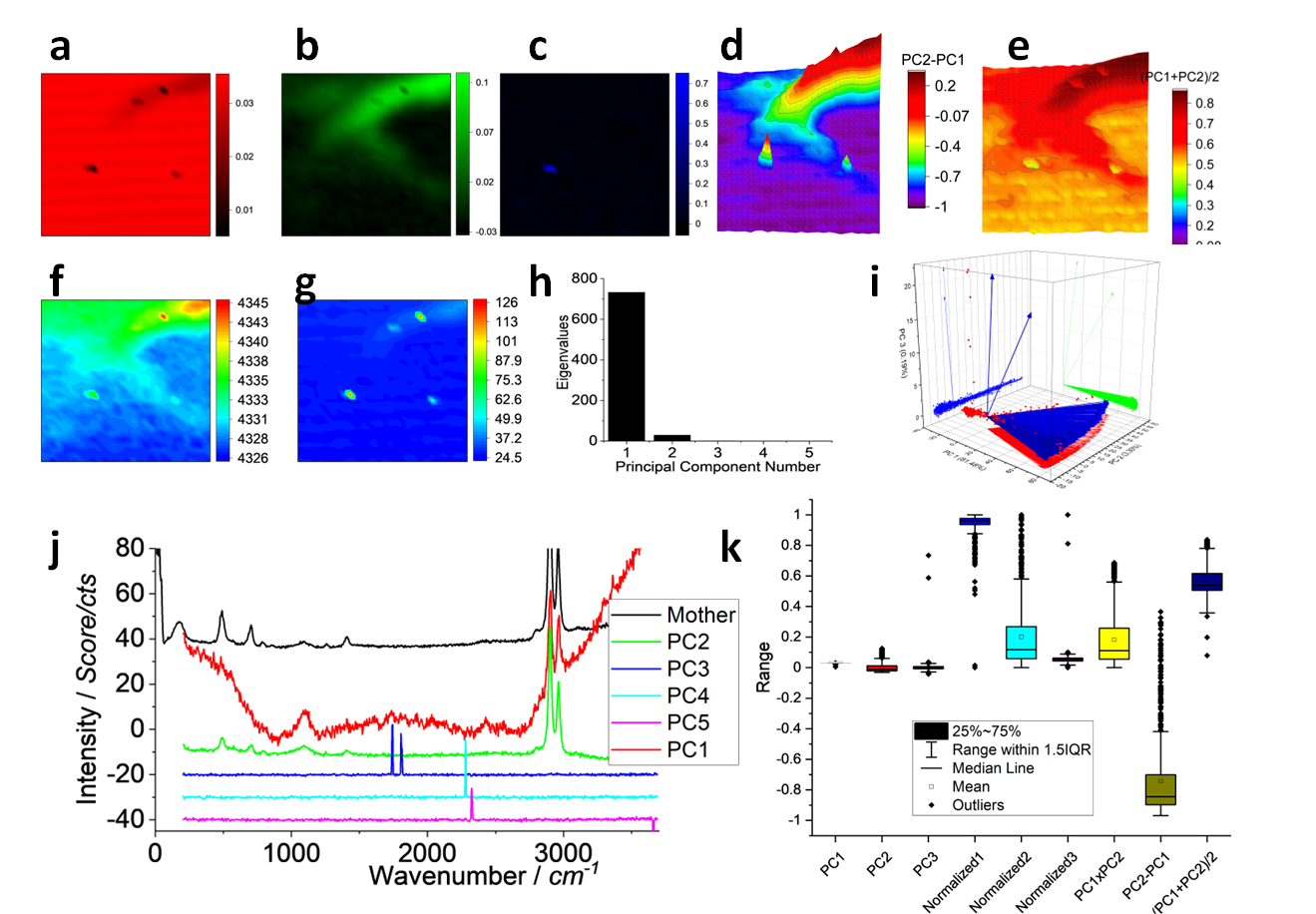


**Figure S8**. PCA analysis parameter for Figure 5. (a-e) are the images mapping the loading coefficients of PC1-PC3 (a-c) and the merged PC1 and PC2 (d-e), using different algebra functions, as indicated in the colour scale bars. (f) is the mapped mean, while (g) the standard deviation of the PCA calculation. (h) is the scree plot and (i) the bi-plot. (j) is the PCA spectrum and (k) shows the distribution ranges of the loading coefficients, including before and after normalisation, and being subjected to merge.

(a-c) map the loading coefficients of PC1-PC3, respectively. (c) shows only one dot or pixel pattern for PC3, which might be due to the cosmic ray. It does thus not take parting the algebra calculation. The different extraction using the algebra functions generate the images (d-e). (f) is the mapped mean to visualise the area where the main signals are collected. (g) implies the areas that we should pay more attention to the analysis results. (h) shows the main variance of the eigenvalues has been taken in PC1. (i) suggests the main PCs, including PC1-PC3, are almost independent from each other again. (j) is the PCA spectra to compare with the mother one. The loading coefficients can be compared in (k).


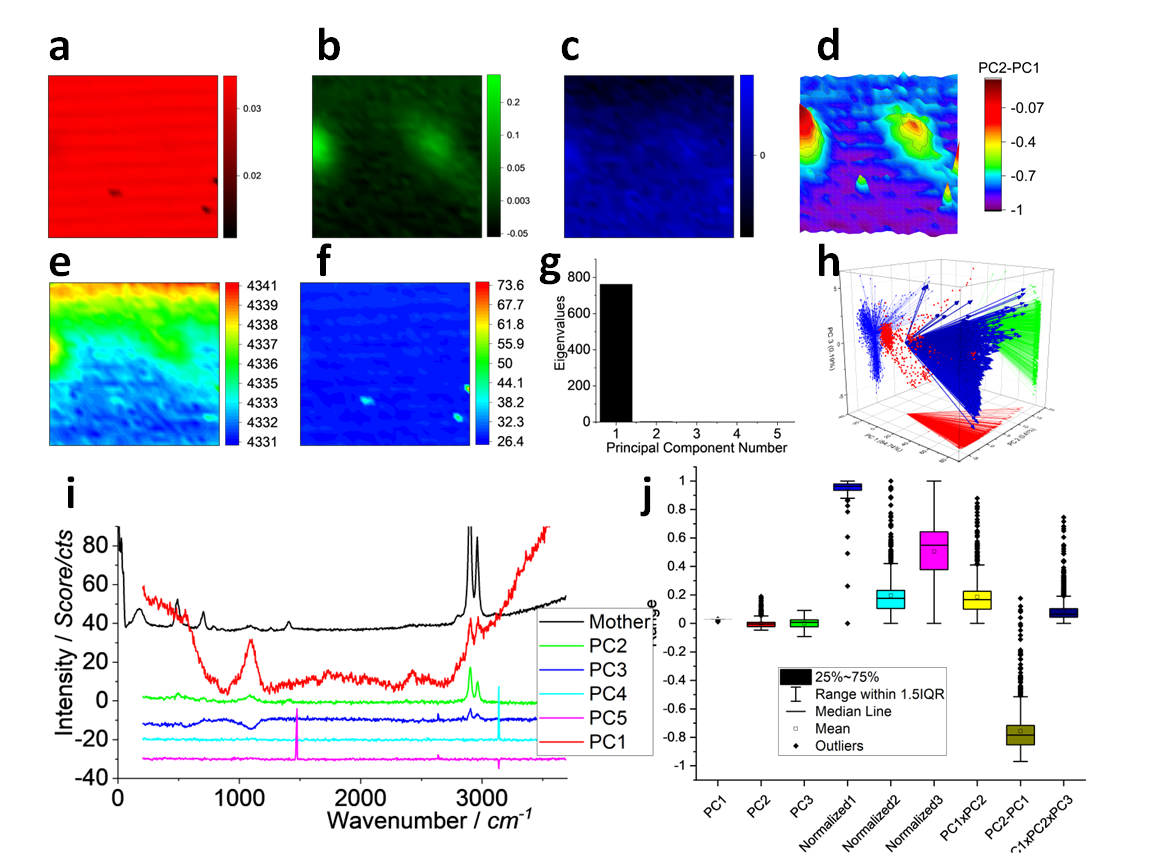


**Figure S9**. PCA analysis parameter for Figure 5. (a-fd are the images mapping the loading coefficients of PC1-PC3 (a-c) and the merged PC1 and PC2 (d), using different algebra functions, as indicated in the colour scale bars. (e) is the mapped mean while (g) the standard deviation of the PCA calculation. (g) is the scree plot and (h) the bi-plot. (i) presents the PCA spectrum and (e) shows the distribution ranges of the loading coefficients, including before and after normalisation, and subjected to merge.

More PCA analysis information is provided herein. Basically the same as above, the merged image can visualise the silicone with an increased certainty.

# Figure S10: more PCA analysis parameters for Figure 6


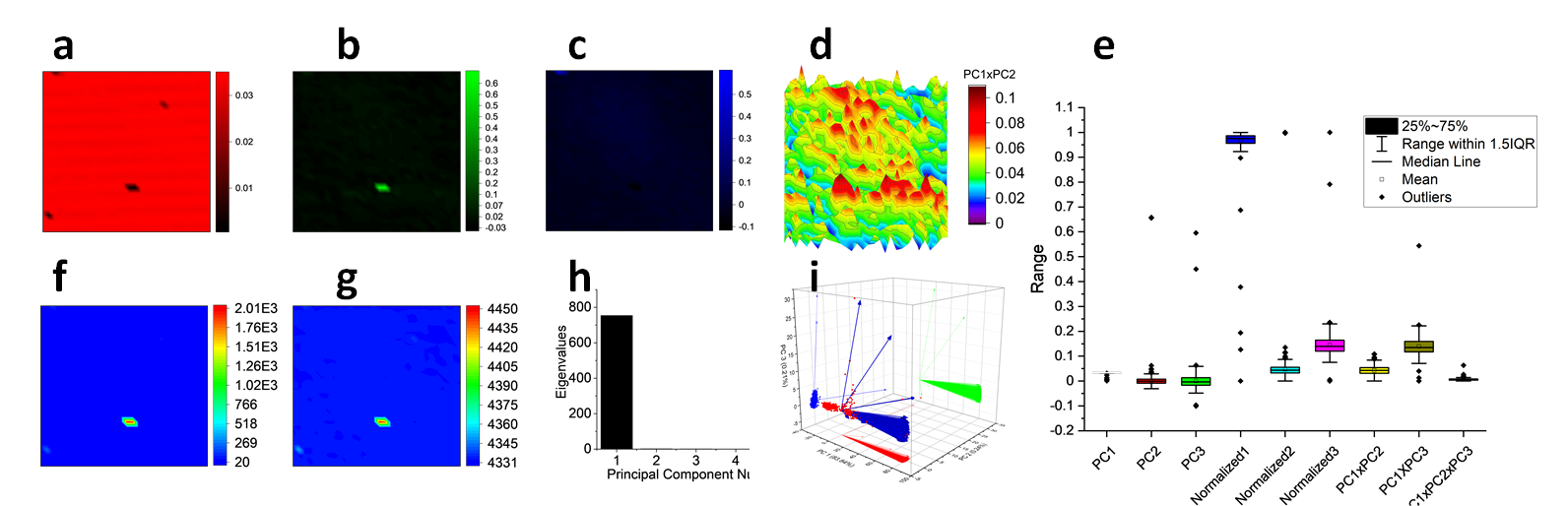


**Figure S10**. PCA analysis parameter for Figure 6. (a-d) are the images mapping the loading coefficients of PC1-PC3 (a-c) and the merged PC1 and PC2 (d), using different algebra functions, as indicated in the colour scale bar. (e) shows the distribution ranges of the loading coefficients, including before and after normalisation, and subjected to merge. (f) is the mapped mean while (g) the standard deviation of the PCA calculation. (h) is the scree plot and (i) the bi-plot.

More PCA analysis information is provided herein. Basically the same as above, the merged image can visualise the silicone with an increased certainty.

# Figure S11 / Tables S1-S2: more fitting analysis parameters for Figure 6


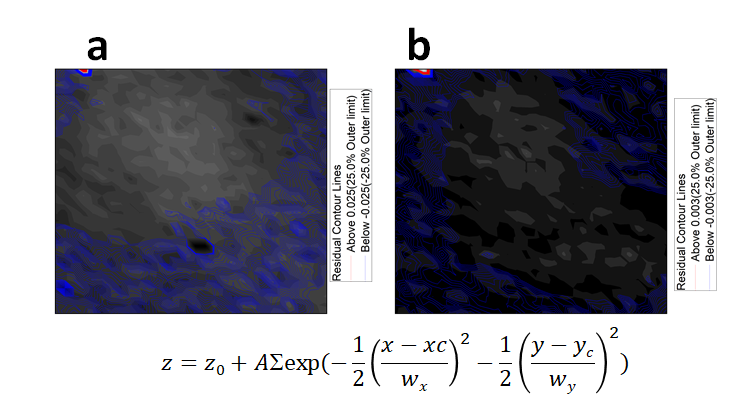


**Figure S11**. Fitting residues for Figures 6(e, g), respectively. The fitting equation is presented as well.

For 2D Gaussian surface fitting, the fitting data residues are shown here. We can see the well match between the raw data and the fitting data. The detailed parameters are listed in the following tables.

Table S1. Fitting Summary for Figure 6(e). (all data for the x × y axis of 30 × 30)

|  | z0 | | A | | xc | | w1 | | yc | | w2 | | | FWHMx | | FWHMy | | |
| --- | --- | --- | --- | --- | --- | --- | --- | --- | --- | --- | --- | --- | --- | --- | --- | --- | --- | --- |
|  | Value | Standard Error | Value | Standard Error | Value | Standard Error | Value | Standard Error | Value | Standard Error | | Value | Standard Error | Value | Standard Error | Value | Standard Error |  |
| Peak1(PC1XPC3) | 0.10971 | -- | 0.24693 | -- | 3.86608 | -- | 1.14193 | -- | 29.51953 | -- | | 0.32517 | -- | 2.68903 | 0 | 0.76572 | 0 |  |
| Peak2(PC1XPC3) | 0.10971 | -- | 0.09839 | -- | 14.22011 | -- | 10.93439 | -- | 19.75169 | -- | | 10.08092 | -- | 25.74853 | 0 | 23.73875 | 0 |  |
| Peak3(PC1XPC3) | 0.10971 | -- | 0.09333 | -- | 13.80213 | -- | 13.70469 | -- | 22.65664 | -- | | 12.63499 | -- | 32.27208 | 0 | 29.75312 | 0 |  |
| Peak4(PC1XPC3) | 0.10971 | -- | 0.09278 | -- | 13.81258 | -- | 14.16235 | -- | 18.90951 | -- | | 13.05693 | -- | 33.34979 | 0 | 30.74671 | 0 |  |
| Peak5(PC1XPC3) | 0.10971 | -- | 0.08172 | -- | 9.43518 | -- | 3.21601 | -- | 21.06904 | -- | | 2.13204 | -- | 7.57312 | 0 | 5.02057 | 0 |  |
| Peak6(PC1XPC3) | 0.10971 | -- | 0.07188 | -- | 7.99175 | -- | 2.95865 | -- | 28.76225 | -- | | 2.33948 | -- | 6.96708 | 0 | 5.50906 | 0 |  |
| Peak7(PC1XPC3) | 0.10971 | -- | 0.06885 | -- | 19.68961 | -- | 2.1305 | -- | 17.12424 | -- | | 1.44286 | -- | 5.01694 | 0 | 3.39767 | 0 |  |
| Peak8(PC1XPC3) | 0.10971 | -- | 0.06561 | -- | 12.75256 | -- | 3.15423 | -- | 27.93053 | -- | | 1.99642 | -- | 7.42763 | 0 | 4.70122 | 0 |  |
| Peak9(PC1XPC3) | 0.10971 | -- | 0.04669 | -- | 5.66959 | -- | 4.84803 | -- | 17.48114 | -- | | 3.25534 | -- | 11.41625 | 0 | 7.66575 | 0 |  |
| Peak10(PC1XPC3) | 0.10971 | -- | 0.04388 | -- | 20.50576 | -- | 2.96989 | -- | 4.6926 | -- | | 1.7584 | -- | 6.99355 | 0 | 4.14072 | 0 |  |
| Peak11(PC1XPC3) | 0.10971 | -- | 0.03913 | -- | 23.82416 | -- | 3.20087 | -- | 5.16608 | -- | | 2.55273 | -- | 7.53747 | 0 | 6.01123 | 0 |  |
| Peak12(PC1XPC3) | 0.10971 | -- | 0.03567 | -- | 4.90449 | -- | 2.83124 | -- | 13.99738 | -- | | 2.53608 | -- | 6.66707 | 0 | 5.972 | 0 |  |
| Peak13(PC1XPC3) | 0.10971 | -- | 0.02686 | -- | 21.56544 | -- | 1.54229 | -- | 1.98803 | -- | | 1.02334 | -- | 3.63181 | 0 | 2.40978 | 0 |  |
| Peak14(PC1XPC3) | 0.10971 | -- | 0.0266 | -- | 9.65568 | -- | 1.64504 | -- | 5.9672 | -- | | 0.81002 | -- | 3.87378 | 0 | 1.90744 | 0 |  |
| Peak15(PC1XPC3) | 0.10971 | -- | 0.02634 | -- | 7.46951 | -- | 3.62878 | -- | 14.04552 | -- | | 2.51881 | -- | 8.54513 | 0 | 5.93135 | 0 |  |
| Peak16(PC1XPC3) | 0.10971 | -- | 0.01831 | -- | 1.72986 | -- | 1.08352 | -- | 8.9361 | -- | | 0.88827 | -- | 2.55149 | 0 | 2.09172 | 0 |  |
| Peak17(PC1XPC3) | 0.10971 | -- | 0.0181 | -- | 24.62633 | -- | 2.08228 | -- | 27.86375 | -- | | 1.46277 | -- | 4.9034 | 0 | 3.44456 | 0 |  |
| Peak18(PC1XPC3) | 0.10971 | -- | -- | -- | -- | -- | 0 | -- | -- | -- | | 0 | -- | 0 | 0 | 0 | 0 | -- |

Table S2. Fitting Summary for Figure 6(g). (all data for the x × y axis of 30 × 30)

|  | z0 | | | A | | xc | | w1 | | yc | | w2 | | FWHMx | | | FWHMy | | |
| --- | --- | --- | --- | --- | --- | --- | --- | --- | --- | --- | --- | --- | --- | --- | --- | --- | --- | --- | --- |
|  | Value | Standard Error | Value | | Standard Error | Value | Standard Error | Value | Standard Error | Value | Standard Error | Value | Standard Error | | Value | Standard Error | Value | Standard Error |  |
| Peak1(PC1xPC2xPC3) | 0.00188 | -- | 0.01661 | | -- | 3.57437 | -- | 1.20018 | -- | 29.35669 | -- | 0.66024 | -- | | 2.82622 | 0 | 1.55474 | 0 |  |
| Peak2(PC1xPC2xPC3) | 0.00188 | -- | 0.0133 | | -- | 11.80416 | -- | 4.81653 | -- | 25.58687 | -- | 4.90838 | -- | | 11.34207 | 0 | 11.55836 | 0 |  |
| Peak3(PC1xPC2xPC3) | 0.00188 | -- | 0.01267 | | -- | 12.5433 | -- | 5.42128 | -- | 22.58433 | -- | 5.52466 | -- | | 12.76614 | 0 | 13.00958 | 0 |  |
| Peak4(PC1xPC2xPC3) | 0.00188 | -- | 0.01199 | | -- | 13.31075 | -- | 4.56335 | -- | 19.64571 | -- | 3.8843 | -- | | 10.74586 | 0 | 9.14683 | 0 |  |
| Peak5(PC1xPC2xPC3) | 0.00188 | -- | 0.01074 | | -- | 16.03985 | -- | 2.74255 | -- | 17.58484 | -- | 3.1474 | -- | | 6.45822 | 0 | 7.41155 | 0 |  |
| Peak6(PC1xPC2xPC3) | 0.00188 | -- | 0.01069 | | -- | 10.48301 | -- | 5.90905 | -- | 19.01249 | -- | 5.02976 | -- | | 13.91475 | 0 | 11.84418 | 0 |  |
| Peak7(PC1xPC2xPC3) | 0.00188 | -- | 0.0098 | | -- | 14.31635 | -- | 4.48353 | -- | 12.41421 | -- | 1.83807 | -- | | 10.55791 | 0 | 4.32833 | 0 |  |
| Peak8(PC1xPC2xPC3) | 0.00188 | -- | 0.00972 | | -- | 18.96542 | -- | 6.47109 | -- | 8.27222 | -- | 5.52853 | -- | | 15.23826 | 0 | 13.01868 | 0 |  |
| Peak9(PC1xPC2xPC3) | 0.00188 | -- | 0.00897 | | -- | 22.97649 | -- | 2.67355 | -- | 12.7308 | -- | 1.79905 | -- | | 6.29573 | 0 | 4.23644 | 0 |  |
| Peak10(PC1xPC2xPC3) | 0.00188 | -- | 0.00877 | | -- | 24.45642 | -- | 7.06584 | -- | 7.01767 | -- | 6.03664 | -- | | 16.63877 | 0 | 14.2152 | 0 |  |
| Peak11(PC1xPC2xPC3) | 0.00188 | -- | 0.00842 | | -- | 11.03876 | -- | 2.60006 | -- | 8.00532 | -- | 1.20889 | -- | | 6.12267 | 0 | 2.84671 | 0 |  |
| Peak12(PC1xPC2xPC3) | 0.00188 | -- | 0.0079 | | -- | 7.5464 | -- | 2.12503 | -- | 29.37449 | -- | 1.57233 | -- | | 5.00407 | 0 | 3.70256 | 0 |  |
| Peak13(PC1xPC2xPC3) | 0.00188 | -- | 0.00777 | | -- | 17.32133 | -- | 2.16894 | -- | 7.07073 | -- | 1.56623 | -- | | 5.10746 | 0 | 3.68819 | 0 |  |
| Peak14(PC1xPC2xPC3) | 0.00188 | -- | 0.00761 | | -- | 22.0125 | -- | 8.23046 | -- | 8.48627 | -- | 7.03163 | -- | | 19.38126 | 0 | 16.55822 | 0 |  |
| Peak15(PC1xPC2xPC3) | 0.00188 | -- | 0.00747 | | -- | 27.91627 | -- | 8.43918 | -- | 5.82699 | -- | 7.20994 | -- | | 19.87275 | 0 | 16.97812 | 0 |  |
| Peak16(PC1xPC2xPC3) | 0.00188 | -- | 0.00638 | | -- | 27.15011 | -- | 4.34723 | -- | 11.13761 | -- | 3.2457 | -- | | 10.23695 | 0 | 7.64303 | 0 |  |
| Peak17(PC1xPC2xPC3) | 0.00188 | -- | 0.00612 | | -- | 15.99273 | -- | 1.14603 | -- | 28.70304 | -- | 2.03862 | -- | | 2.69869 | 0 | 4.80058 | 0 |  |
| Peak18(PC1xPC2xPC3) | 0.00188 | -- | 0.00556 | | -- | 17.01525 | -- | 2.48529 | -- | 27.24041 | -- | 3.84029 | -- | | 5.8524 | 0 | 9.04318 | 0 |  |
| Peak19(PC1xPC2xPC3) | 0.00188 | -- | 0.0053 | | -- | 26.48807 | -- | 6.06572 | -- | 10.58198 | -- | 4.52874 | -- | | 14.28368 | 0 | 10.66437 | 0 |  |
| Peak20(PC1xPC2xPC3) | 0.00188 | -- | 0.00522 | | -- | 8.05592 | -- | 2.53243 | -- | 16.68753 | -- | 1.50898 | -- | | 5.96341 | 0 | 3.55337 | 0 |  |
| Peak21(PC1xPC2xPC3) | 0.00188 | -- | 0.005 | | -- | 10.88934 | -- | 3.69482 | -- | 11.47204 | -- | 3.31887 | -- | | 8.70064 | 0 | 7.81535 | 0 |  |
| Peak22(PC1xPC2xPC3) | 0.00188 | -- | 0.00499 | | -- | 4.46028 | -- | 3.61005 | -- | 7.36586 | -- | 4.33291 | -- | | 8.50101 | 0 | 10.20323 | 0 |  |
| Peak23(PC1xPC2xPC3) | 0.00188 | -- | 0.00406 | | -- | 28.11487 | -- | 5.06035 | -- | 28.53755 | -- | 4.5888 | -- | | 11.91621 | 0 | 10.8058 | 0 |  |
| Peak24(PC1xPC2xPC3) | 0.00188 | -- | 0.00398 | | -- | 6.8572 | -- | 5.9163 | -- | 10.36189 | -- | 2.85462 | -- | | 13.93182 | 0 | 6.72212 | 0 |  |
| Peak25(PC1xPC2xPC3) | 0.00188 | -- | 0.00382 | | -- | 7.65129 | -- | 4.3011 | -- | 3.04685 | -- | 5.16234 | -- | | 10.12831 | 0 | 12.15637 | 0 |  |
| Peak26(PC1xPC2xPC3) | 0.00188 | -- | 0.00376 | | -- | 26.08549 | -- | 5.36401 | -- | 26.63465 | -- | 4.86417 | -- | | 12.63128 | 0 | 11.45423 | 0 |  |
| Peak27(PC1xPC2xPC3) | 0.00188 | -- | 0.00362 | | -- | 1.9046 | -- | 17.79733 | -- | 25.41284 | -- | 9.79169 | -- | | 41.90951 | 0 | 23.05767 | 0 |  |
| Peak28(PC1xPC2xPC3) | 0.00188 | -- | 0.00317 | | -- | 27.15984 | -- | 7.67328 | -- | 16.8558 | -- | 9.4533 | -- | | 18.06918 | 0 | 22.26082 | 0 |  |
| Peak29(PC1xPC2xPC3) | 0.00188 | -- | 0.00307 | | -- | 3.31493 | -- | 5.28959 | -- | 4.01787 | -- | 6.34876 | -- | | 12.45602 | 0 | 14.95018 | 0 |  |
| Peak30(PC1xPC2xPC3) | 0.00188 | -- | 0.00216 | | -- | 29.13716 | -- | 16.11185 | -- | 25.29143 | -- | 14.61047 | -- | | 37.94051 | 0 | 34.40503 | 0 |  |
| Peak31(PC1xPC2xPC3) | 0.00188 | -- | -- | | -- | -- | -- | 0 | -- | -- | -- | 0 | -- | | 0 | 0 | 0 | 0 |  |

# Figure S12: real roof sample


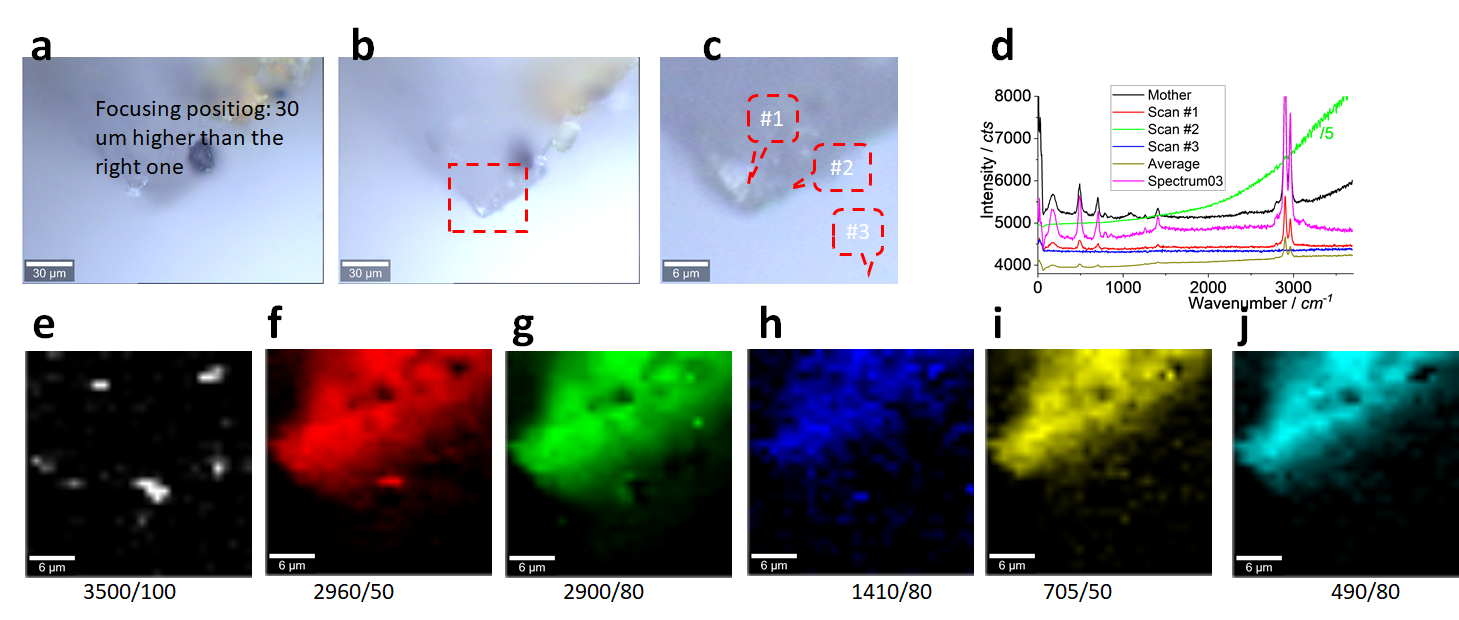


**Figure S12**. Photo images (a-c), typical Raman spectra (d) and Raman intensity images (e-j). The sample was directly collected from a roof where the silicone has been applied for ~7 years. The focusing position is different between (a) and (b), with ~30 μm along *z-*axis. The area in (b) of 30 μm × 30 μm was zoomed in as (c) and scanned. Raman spectra were collected under an objective lens of 100×, integration time of 1 s for each pixel of 1 μm × 1 μm (to create a matrix of 30 × 30). (d) shows the Raman spectra of the mother silicone, to compare with 1 single spectrum (#03) (10 s) and 3 typical scanning spectra (1 s) collected from the marked positions in (c), and their average spectrum of 900 (30 × 30) spectra. The intensity images (e-j) are mapped at a blank wavenumber window (e), the characteristic peaks of PC (f-j), as marked under each image (and the peak width), after 10% colour off-setting.

Herein for comparison, we also tested the roof silicone. The real sample is tested in this section and the mimicked sample is presented in the next section. Basically, the real sample is more complicated than the mimicked sample, such as the high spectrum background, particularly for Scan#2. Even so, the Raman imaging still can visualise the silicone and suggest the presence.

For confocal Raman, the signal is effectively collected from the focal along the *z-*axis. Off-focal position cannot be effectively imaged. This is shown in (a, b), that photos at different focused positions along *z-*axis. That is also the reason why only part of the particle is imaged in (f-j), where the particle is focused along the *z-*axis.

# Figure S13: mimicked roof sample


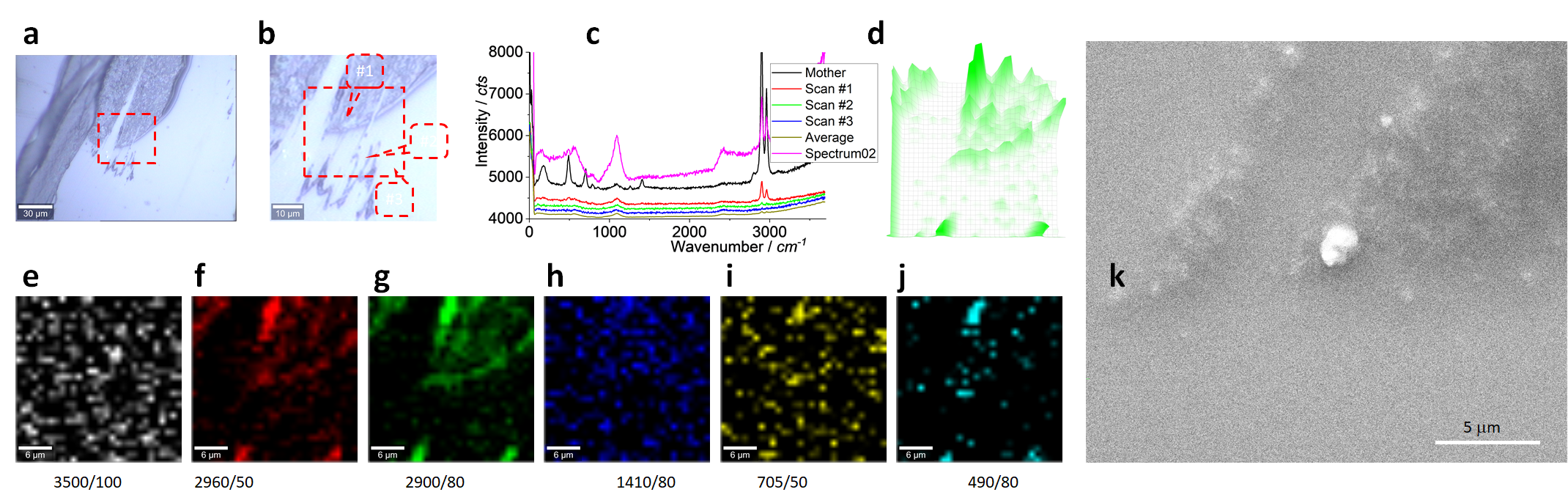


**Figure S13**. Photo images (a-b), typical Raman spectra (c) and Raman intensity images (d-j). The sample was collected from mimicked roof on glass slide. The area in (a) of 30 μm × 30 μm was zoomed in as (b) and scanned. Raman spectra were collected under an objective lens of 100×, integration time of 1 s for each pixel of 1 μm × 1 μm (to create a matrix of 30 × 30). (c) shows the Raman spectra of the mother silicone, to compare with 1 single spectrum (#02) (10 s) and 3 typical scanning spectra (1 s) collected from the marked positions in (b), and their average spectrum of 900 (30 × 30) spectra. The intensity images (d-j) are mapped at a blank wavenumber window (d), the characteristic peaks of PC (e-j), as marked under each image (and the peak width), after 10% colour off-setting.

The mimicked sample is a little different from the real sample. However, we still can assign the pattern to visualise silicone, although some images are blurred, due to the weak peaks.
